# Using clinical supervision to improve the quality and safety of patient care: a response to Berwick and Francis

**DOI:** 10.1186/s12909-015-0324-3

**Published:** 2015-06-11

**Authors:** Jonathon Tomlinson

**Affiliations:** NIHR In Practice research fellow, Centre for Primary Care and Public Health, Barts and the London School of Medicine and Dentistry, London, E1 2AB UK

**Keywords:** Supervision, Narrative, Quality, Safety, Reflection, Education, Coaching, Mentoring

## Abstract

**Background:**

After widely publicised investigations into excess patient deaths at Mid Staffordshire hospital the UK government commissioned reports from Robert Francis QC and Professor Don Berwick. Among their recommendations to improve the quality and safety of patient care were lifelong learning, professional support and ‘just culture’. Clinical supervision is in an excellent position to support these activities but opportunities are in danger of being squeezed out by regulatory and managerial demands. Doctors who have completed their training are responsible for complex professional judgements for which narrative supervision is particularly helpful. With reference to the literature and my own practice I propose that all practicing clinicians should have regular clinical supervision.

**Discussion:**

Clinical supervision has *patient-safety and the quality of patient care as its primary purposes*. After training is completed, doctors may practice for the rest of their career without any clinical supervision, the implication being that the difficulties dealt with in clinical supervision are no longer difficulties, or are better dealt with some other way. Clinical supervision is sufficiently flexible to be adapted to the needs of experienced clinicians as its forms can be varied, though its functions remain focused on patient safety, good quality clinical care and professional wellbeing.

**Summary:**

The evidence linking clinical supervision to the quality and safety of patient care reveals that supervision is most effective when its educational and supportive functions are separated from its managerial and evaluative functions. Among supervision’s different forms, narrative-based-supervision is particularly useful as it has been developed for clinicians who have completed their training. It provides ways to explore the complexity of clinical judgements and encourages doctors to question one another’s authority in a supportive culture. To be successful, supervision should also be professionally led and learner centred rather than externally imposed and centred on institutions. I propose that regular clinical supervision should be a professional requirement if the quality and safety aspirations of Francis and Berwick are to be met.

## Background

In the wake of serious patient harm at Mid Staffordshire hospital and the Francis Report [1] the government commissioned Professor Don Berwick to lead a National Patient Safety Advisory Group tasked with making ‘zero harm a reality in our NHS’. The first and over-riding recommendation of the Berwick report [2] was for,

*… [the NHS] to become, more than ever before, a system devoted to continual learning and improvement of patient care, top to bottom and end to end.*

The report concludes,

*The most powerful foundation for advancing patient safety in the NHS lies much more in its potential to be a learning organisation, than in the top down mechanistic imposition of rules, incentives and regulations.*

Guidance from the General Medical Council (GMC) states,

*All members of the multi-professional team (doctors, senior nurses and allied health professionals) are involved in clinical supervision to less experienced doctors as part of their clinical job and professional duty to ensure patients receive safe and quality patient care* [3]*.*

The Royal College of General Practitioners (RCGP) 2014 curriculum statement is clear,

*You [GPs] will also need to play a role in the personal and professional development of others through activities such as coaching, mentoring and supervision.*

Healthcare professionals are continually learning when they are at work and a great deal of this learning comprises core features of clinical supervision. Francis gives a very favourable description ^(vol.32.57)^ of educational and supportive clinical supervision at St Christopher’s hospice where staff had daily opportunities to train and reflect on their training together, hierarchies were flattened and role modelling encouraged.

## Discussion

### A working definition of clinical supervision

If clinical supervision is to be recommended, we need some clarity about what it entails.

Because its forms are so varied, it is usually defined in terms of three functions, based on Proctor’s Functional Interactive Model [4]: normative (managerial), formative (educational) and restorative (supportive). Milne [5] proposed a fourth, evaluative function and claimed that clinical supervision without evaluation was coaching or mentoring.

In contrast to Milne, the independent regulator of health and social care services in England, the Care Quality Commission (CQC) [6] claims that clinical and professional supervision do *not* include an evaluative component, being *separate from managerial supervision, which is about monitoring and appraising the performance of staff.* The reference guides for postgraduate specialist and general practice training in the UK do not define clinical supervision, but describe its functions in terms of overseeing clinical work, providing constructive feedback, and teaching the need for safer practice and better patient care [7,8]. The Association of Medical Educators (AoME) Guidance for Deaneries on which the GMC guidance for recognition and approval of trainers draws on, also describes its functions, stating that clinical supervisors are responsible for, *1. Ensuring safe and effective patient care through training, 2. Establishing and maintaining an environment for learning, 3. Teaching and facilitating learning, 4. Enhancing and learning through assessment* and *5. Continuing professional development as an educator* [9]*.*

Kilminster et al. [10] defined clinical supervision in the context of trainees in 2007, but their definition from 2000 refers to doctors rather than trainees,

*[We define clinical supervision as] The provision of monitoring, guidance and feedback on matters of personal, professional and educational development in the context of the doctor’s care of patients. This would include the ability to anticipate a doctor’s strengths and weaknesses in particular clinical situations in order to maximize patient safety* [11].

They do not explain why they later replaced ‘doctors’ with ‘trainees’ but it may be an acknowledgment that consultants and GPs tend not to have any supervision. The GMC includes a section on maintaining performance under the Duties of a Doctor, stating, *you should be willing to find and take part in structured support opportunities offered by your employer or contracting body (for example mentoring).* And, *You must make sure that all staff you manage have appropriate supervision* [12]*.*

‘Narrative based supervision’, also called ‘Conversations Inviting Change’ shares the same dialogic questioning that characterises narrative based medicine from which it was developed [13,14]. The story, told by the patient or supervisee, is re-created and re-interpreted by the questions asked by the listener – doctor or supervisor, and in so doing, underlying assumptions and interpretations are challenged [15,16]. Many forms of supervision share narrative-based supervision’s questioning to different extents [17,18]. Informal conversations between colleagues, over coffee or in a corridor, may be little more than friendly words of advice. If it is supervision that is wanted or being offered, then this should be made explicit, because it will be a different kind of more serious, questioning conversation. Undrill warns that even if these conversations take on a supervisory nature, they are *no substitute for properly contracted, regular supervision* [17].

What all these descriptions, definitions and guidelines have in common is that clinical supervision is a questioning learning activity that is focussed on clinical work, involves clinicians teaching one-another and is collaborative, benefitting both supervisors and supervisees. It is also usually on-going, allowing relationships to develop, and reflective, nurturing insight and critical thinking, and supportive, building resilience and wellbeing.

### Appraisal and revalidation

For NHS consultants and GPs who have completed their training, an annual appraisal which forms the basis of evidence for revalidation may be their only experience of supervision [19]. Appraisals are an aspect of Clinical Governance which focuses on individuals rather than organisational or system level factors.

For GPs, an online appraisal form has to be completed before a one-to-one meeting which lasts 3–5 hours. Most of the appraisal form is focussed on supervision’s managerial function, requiring the assessment of evidence which includes written reflection. This concept of reflection as a solitary activity that is attained and assessed is perhaps a consequence of appraisal’s evaluative emphasis. It is in contrast to the way reflection was developed in medical education as a dialogical activity and a process of discovery [20]. When reflection is mandated and evaluated, the events are not necessarily those that matter because important issues of clinical and ethical uncertainty that expose the doctor's vulnerability are less likely to be explored. By contrast, for example, in Schwartz rounds cases are often chosen for presentation because they reveal emotional difficulty, clinical uncertainty or professional vulnerability. A case is presented, often by more senior professionals to an audience that can include peers, but can include any professional involved in the case, and a discussion about the emotional and relational content is facilitated. Feedback from junior doctors has been that this form of discussion-based, un-assessed reflection is far more valuable than the private, assessed reflection they had been forced to do before. (personal communication with Jocelyn Cornwell, Point of Care Foundation)

GPs’ concerns that the educational and supportive functions which they wanted in appraisals would be squeezed out by the managerial and evaluative functions required by revalidation have persisted from 2003 when they were introduced [21-23]. These are also common complaints among nurses and social workers [24,25]. A recent review paper about clinical supervision recommends that management and evaluation are kept separate from supervision’s other roles [24]. Combining assessment with support leaves doctors in a position of wanting to show off their strengths on which they can be assessed, but not wanting to reveal their weaknesses with which they need support, out of fear that these will be held against them. Doctors who are mentally unwell or ashamed are especially reluctant to discuss their difficulties [26,27].

Killminster et al. emphasise that supervision may *only* be effective when supervisees control the products of supervision [10]. Lacking any other formal time for professional development and support, it is unsurprising that some doctors do find appraisals valuable, but others are intimidated and/ or view it as a bureaucratic and ineffective attempt to spot poor practice and the two viewpoints cannot easily be reconciled [23,28,29]. Appraisals may assess whether clinical supervision has taken place, but do not in themselves allow timely education and support around issues of quality and safety as they arise and cannot substitute for regular clinical supervision. We are required to supplement appraisals with feedback about our colleagues’ clinical judgement and attitudes to patients in order for them to be revalidated, but because we so rarely observe them in practice our judgement may be little more than an expression of how much we like or respect them. A good doctor working as a locum in different practices or within in a dysfunctional team may therefore struggle to be revalidated.

US surgeon and writer, Atul Gawande who is giving this year’s Reith Lectures gave a powerful example of how the most experienced doctors can benefit from coaching in the same way as professional sportsmen and women do [30]. Gawande, an experienced surgeon, was used to operating independently, without supervision, but invited a respected colleague to watch him perform a routine operation. His colleague made several critical observations that made Gawande realise that unless supervision is required or actively solicited, opportunities to improve the quality and safety of patient care will be missed.

**Example of one to one, peer supervision.** B., a salaried GP in our practice, and I recently observed each other during a surgery. We have both had training in clinical supervision and prepared by spending an hour discussing issues of power in the supervisory relationship and identifying areas in the consultation that we wanted to give particular attention to. All the patients were warned before-hand that another doctor would be watching the surgery. We watched each other for the duration of a four hour surgery with about twenty appointments each. Issues that we both identified included different ways we used IT, the language we used to introduce ourselves and give reassurance to patients and the different ways we responded to patient cues. Working in isolation, we realised that we rarely articulate the uncertainty that characterises so much of our work. The limits to how much we can tolerate vary not only due to our knowledge but our emotional resilience. The support we gained from having a colleague with us meant that for both of us there were patients who might otherwise have been referred for a hospital specialist opinion or an ‘unnecessary’ investigation. B had asked me to watch for how she concluded consultations, as she was concerned that her patients were coming frequently for reassurance and she was often running late. We were able to identify examples where, in trying to reassure patients she was explaining details for her own reassurance rather than responding to patient cues and giving them confidence to manage safely themselves. Watching me, she noticed an occasion where I colluded with a patient’s interpretation about her symptoms being caused by her housing situation. She challenged my approach, helping me to think about other ways of consulting that could maintain a delicate therapeutic relationship whilst challenging patients.We both found the experience so useful that we have agreed to repeat it in future using our study leave allowance to make time.

### Narrative supervision and patient safety

The most powerful and influential aspects of the Francis Enquiry into Mid Staffordshire hospital were the patient and relative narratives which were quoted throughout the report. Robert Francis QC who chaired the inquiry noted their importance [31]*,*

*“I heard so many stories of shocking care,” he said. “They were people who entered Stafford hospital and rightly expected to be well cared for and treated. Instead, many suffered horrific experiences that will haunt them and their loved ones for the rest of their lives.”*

One of his recommendations ^(Vol.3 rec. no. 40)^ was that greater attention be paid to the narrative contained in complaints data.

Analysing narratives is already an established part of patient-safety investigations. For example, the analysis of different narratives from the professionals involved in a routine operation when airline pilot Martin Bromiley’s wife died in 2005 revealed the problems in communication that led to her death [32]. Bromiley founded the Clinical Human Factors Group in 2007 and its key message is the importance of listening to and sharing each-others’ narratives within a ‘just culture’ [33]. In a video of him teaching doctors and nurses, he talks about those who were involved in his wife’s death,

*“You will be pleased to know that they all returned to work… and that is exactly what I wanted. They can spread those very personal lessons on to their colleagues and all of them will be much better clinicians as a result of what happened, of that there’s no doubt.”* [34]

Francis and Berwick both emphasise the importance of a ‘just culture’. A ‘just culture’ is one in which means that members are encouraged to report concerns about safety without fear of blame [35]. Francis quoted Sir Liam Donaldson ^(vol.3,20.97)^

*Honest failure is something that needs to be protected otherwise people will continue to live in fear, will not admit their mistakes and the knowledge to prevent serious harm will be buried with the patient.*

The Berwick report begins,

*‘Abandon blame as a tool, NHS staff are not to blame – in the vast majority of cases it is the systems, procedures, conditions, environment and circumstances they face that lead to patient safety problems.’* [2]

Doctors have a deep-rooted attachment to their professional identity so that when mistakes are made, they feel depressed, guilty and ashamed and are afraid of repercussions [36-38]. A large study into culture and safety in the NHS concluded that patient safety depended on staff feeling supported [39]. Unsurprisingly therefore, when staff are afraid of blame, many errors in healthcare go unreported, further illustrating the conflict between supervision’s educational and supportive functions and its managerial and evaluative functions. Although just one of a wide range of patient-safety measures, narrative supervision helps create a safer culture in which difficulties are discussed, questions asked and hierarchies flattened. It is especially suited to the reflective practice necessary to help work though complex problems which combine ethical, personal and institutional issues [39-41].

### Cultural change and clinical supervision

The theme of Chapter 20 of Francis’ report concerns ‘culture’ and how it can be changed. Culture is a social construct that comprises shared values, assumptions and learning, it is both resilient and in constant flux. Hafferty, Emmerich and Sinclair have all shown that culture, as expressed by doctors’ attitudes, is strongly influenced by the nature of the relationships and rituals they experience at medical school and in practice, the so-called hidden curriculum [42-44]. One of the Francis’ more controversial recommendations, number 185, Focus on the Culture of Caring is,

*Selection of recruits to the profession who evidence the:*

- *Possession of the appropriate values, attitudes and behaviours;*
- *Ability and motivation to enable them to put the welfare of others above their own interests*

Responding to hypothesis that this is both possible and can be sustained beyond the process of recruitment, nursing Professor Jill Maben finds little evidence of either. Instead she shows the vast majority of nurses enter the profession with ideals of altruism and a desire to make a difference, but these were lost, often within months of qualification, as their ideals become compromised or crushed, frequently due to excessive workload combined with a lack of structural and personal support [45,46]. This was acknowledged by Berwick, who said,

*NHS staff are not to blame – in the vast majority of cases it is the systems, procedures, conditions, environment and constraints they face that lead to patient safety problems.*

Alongside better systems, supportive supervision can play a role in creating a safe, supportive culture. Because narrative supervision depends on the quality of the questioning, rather than clinical knowledge or experience, senior clinicians can act as supervisees as in Schwartz rounds and one to one, peer supervision as described above. They can model uncertainty, ethical difficulty, emotional impact, the desire to learn from others and a willingness to be questioned [17]. Less senior doctors can develop the skills and confidence to ask their senior colleagues difficult questions. The more they do this the more likely it is to become habitual. This helps to explain why narrative supervision for Launer, is *part of a wider project to bring about cultural change in medicine* [14]. Role modelling is an important influence of culture. Medical students’ concepts of a ‘good doctor’ and is often challenged by the negative role models they witness in practice [47]. Role models can support or hinder learning about professionalism, patient centred care and empathy [48,49]. Positive role models are good teachers who pay attention to the doctor-patient relationship and concern for the psychological and social aspects of disease [50].

Francis notes that a shared positive culture requires, amongst other things, the empowerment of front-line staff with responsibility and freedom to deliver safe care. Berggren and Severinsson showed that clinical supervision empowered nurses to take responsibility for their decisions, support patients and reflect on difficult cases [51]. In this study, clinical supervision was carried out in groups of four or five nurses for an hour and a half each week, was learner centred, educational and supportive. It had no evaluative or managerial functions. Similar studies involving other healthcare professionals, in which the precise nature of the supervisory process was detailed would be very useful.

Coping with the complexity of clinical practice requires not just competency but capability, the ability – throughout one’s career - to adapt to change, generate new knowledge and continuously improve performance [52]. Capability places greater emphasis on understanding problems than ‘check-list’ approaches *which are useful only once the problem has been understood*. Knowing what *could* be done in a clinical situation can be expressed as guidelines and protocols, but making a decision about what *should* be done requires doctors to understand and take into account unique contexts such as patient values and ethical implications. Values based care can help with this, by integrating patient and professional values with medical evidence [53]. But values need to be explored and the careful, circular questioning that characterises narrative-supervision is ideal for this. Ethically informed, context-dependent clinical decision making is often stressful and questioning can reveal more, rather than less uncertainty as perhaps contradictory contextual factors are bought in to play [54,55].

Educating for capability and clinical judgement requires more shared learning, feedback, reflection and mentoring for which narrative-based clinical supervision is ideally suited [56].

### Resilience and burnout

Clinical supervision helps doctors connect with their peers and develop self-awareness and insight such as has been found lacking in doctors referred to the GMC with fitness to practice concerns [57]. A series of articles [58-60] about the treatment of Doctors who have been referred to the General Medical Council (GMC) is very critical about the lack of support. Clinical supervision has a potential role in both preventing problems and in helping doctors who have been referred and is a requirement for all doctors referred to the Practitioner Health Programme for doctors or dentists with mental health or addiction concerns [61].

In discussing the complexity of good supervision, Launer asks, rhetorically, *Why shouldn’t we conceptualise supervision in medicine as primarily therapeutic in its purpose – not just for the supervisee, but also for the supervisor and hopefully for the patients?* He is careful to state that supervision is not therapy, but there is no clear line where our need for supportive supervision ends and our need for psychotherapy begins [62]. As a supervisor I have helped supervisees who are struggling with serious and upsetting patient complaints, seriously ill relatives, personal illnesses and serious clinical errors, all of which can trigger the kind of breakdown that leads one to seek professional help. Clinical supervision has been shown to reduce burnout and compassion fatigue in GPs and increase engagement and empowerment in hospital doctors [63-65]. As a supervisee, particularly one with a responsibility for dealing with patient complaints at my practice, I have found supervision from my peers highly supportive.

**Example of Balint group supervision with experienced GPs.** I meet with a group of six GPs every three weeks for three hours on a Sunday evening. We have been meeting for the last 13 years. Many GPs attend similar groups, but they are not required to do so, and they may meet for educational or other activities that are not supervisory. Every second meeting we discuss clinical cases using narrative supervisory techniques and approximately every sixth session we use Balint Group techniques to discuss difficult cases. A Balint Group is ideal for a group setting because the supervisor is the group. The GP bringing a case for a Balint Group presents the case to their colleagues, explaining the context and their difficulties and the areas they want help with. The other members of the group are given a few minutes to ask questions to ensure they have enough information to discuss the case. The GP who presented the case then sits back from the conversation and the other doctors discuss the case without them contributing or interrupting, though they are allowed to listen to the discussion. The group may have a leader/ facilitator but over years of experience we do not feel the need to have one. We discuss our reactions to the case, how it made us feel, what we might have felt if we were the doctor or the patient and we explore different aspects of the relationship between the doctor and their patient. The emphasis is on the nature of the relationships rather than on clinical knowledge. After the discussion has continued for about twenty minutes the doctor who presented is invited back into the group to respond to the discussion. Almost always the group identifies emotions or contextual factors that were impacting on the case in ways that had not been identified that help explain why the case is causing problems. We may identify important gaps in our knowledge about the patient that might help. Because a Balint group is focused on relationships, it can help support relationship-based, patient-focused care that characterises general practice [66]. The group is very supportive for its members which adds to limited evidence to show that it can reduce burnout [67].

The poor nursing care of elderly patients with dementia was one of the main concerns at Mid Staffs. Whilst supervision cannot substitute for adequate staffing levels or the failure at an organisational level to solicit feedback and respond to concerns, there is evidence that it helps to improve care and reduce professional burnout when nurses are caring for elderly demented patients [68]. Healthcare, especially for nurses is ‘emotional labour’ [69] as Iona Heath notes in her review of Intelligent Kindness,

*It is easy to forget the appalling nature of some of the jobs carried out by NHS staff day in, day out—the damage, the pain, the mess they encounter, the sheer stench of diseased human flesh and its waste products* [70]*.*

Schwartz rounds, described above have been shown to reduce professional isolation and have a positive effect on patient care and organisational culture [71]. A large new study of their effects in the NHS is underway.

### Supervision for all, the problems of access and acceptability

Supervision is not compulsory for doctors after they have finished their training, so that they might spend forty years or more without having any supervision other than their annual appraisal. In my own GP surgery all the salaried doctors are offered and accept an hour of clinical supervision a month with one of the partners, and I am now receiving supervision myself. I also teach the salaried doctors and medical students how to develop their own supervisory skills. All the members of our practice clinical team meet once a week for two hours and part of the meeting is set aside to provide supervision for clinicians with difficult cases. We provide paid, protected time to do this. I know many GPs who do not have this level of supervision but wish that they did. Access to supervision depends on both the willingness of the institution – GP surgery or hospital and the motivation of the individual for it to happen. Almost all of the literature I have referred to notes the importance of protected time if clinical supervision is to happen as the experience of many of my colleagues is that time for supervision is often squeezed out by administrative and regulatory bureaucracy. Francis^(vol.3p,1754)^ says, *it is imperative that … there is time provided for organisations to learn, focus and improve …* A supervision contract, venue, agenda, and feedback mechanisms are all important to ensure supervision takes place [24]. Most supervision is undertaken face to face, but digital technology including Skype and Facetime can be useful in remote areas. Secure social media including Facebook and Wikispaces can be used for both synchronous and asynchronous supervisory activities, though supervisees generally prefer face to face activities [24].

Supervisory skills cannot be taken for granted by virtue of experience and training is necessary to ensure that supervisors are skilled and that there are enough of them for every doctor to benefit for the duration of their career.

### Acceptability

Even if we solve the problem of access, for some doctors, needing to have clinical supervision is thought to be a sign of weakness [59]. Others view private reflection, for example writing a diary or blog or reading literature as more effective. The risks of solitary reflection are that it is either excessively or insufficiently self-critical as McIntyre and Propper stated in 1983,

*We must recognise that self-criticism is best but that criticism by others is necessary and especially valuable if they approach problems from a different background. We must therefore learn to accept gracefully, and even gratefully, criticism from those who draw our attention to our errors* [72]*.*

If clinical supervision is to be made more acceptable, we need to be sure what we mean by it. The evidence I have presented, overwhelmingly supports supervision which is professionally led, learner-centred, educational and supportive. Narrative supervision is particularly suited to more experienced clinicians. Supervision that is externally imposed, institution-centred, managerial and evaluative is less likely to be acceptable or successful [24]. Evidence about its benefits aligned with professionals’ intrinsic motivation such as patient safety, clinical competence, good communication, good teaching and respect for colleagues and patients will all help improve acceptability [47].

### Limitations

Clinical supervision is that part of good clinical governance that applies to individuals and small groups and cannot satisfy Berwick and Francis’ organisational or system-level recommendations. Whilst necessary, it is not sufficient for patient safety, quality care and professional wellbeing. Evidence of its benefits comes from medical, nursing and other professions and it is not clear to what extent it can be applied across disciplines. It has been studied mostly in relation to trainees, but much less with regard to consultants and GPs. Because its forms and outcomes are so varied, studies are very difficult to compare. Some studies, such as those I have discussed in relation to safety and resilience look at the effects of interventions that can be described as clinical supervision even though the authors have not used that term. Many studies are very small, there are very few about narrative supervision and very few have been repeated. Further research should describe the techniques that are used in different forms of supervision, for example, the contribution of narrative supervision to Schwartz-rounds. It would be helpful to have more studies look at the impact of supervision on organisational culture. Action research shares some of the principles of clinical supervision including an emphasis on relationships and time for reflection at the organisational level and has potential to improve engagement where clinical supervision may reinforce a sense of powerlessness and disengagement [73].

Some doctors will resent the emphasis on their own behaviour rather than structural or organisational factors, such as a lack of staffing, poor management and organisational upheaval. It can only go so far without manageable workloads, managerial expertise and political competence.

## Summary

Clinical supervision, especially when it is professionally led, learner-centred, educational and supportive, has the potential go a long way to fulfilling many of the recommendations of the Francis and Berwick reports. In particular it fosters a culture that is educational, self-critical, outward-looking and patient-focussed, centred on patient safety and quality care. Narrative supervision is especially suited to creating an open, questioning, supportive culture. The challenges are to separate out its educational and supportive functions while improving access and acceptability so that it is taken up by every doctor for the duration of their career. These challenges are significant but certainly not insurmountable and the evidence strongly supports this goal.
